# Protease-activated receptors in the Achilles tendon–a potential explanation for the excessive pain signalling in tendinopathy

**DOI:** 10.1186/s12990-015-0007-4

**Published:** 2015-03-17

**Authors:** Jens Christensen, Håkan Alfredson, Gustav Andersson

**Affiliations:** Department of Integrative Medical Biology, Section for Anatomy, Umeå University, Umeå, SE-90187 Sweden; Department of Community Medicine and Rehabilitation, Sports Medicine, Umeå University, Umeå, SE-90187 Sweden; ISEH, UCLH, London, UK; Pure Sports Clinic, London, UK; Department of Surgical and Perioperative Science, Section for Hand and Plastic Surgery, Umeå University, Umeå, SE-90187 Sweden

**Keywords:** Achilles tendon, Protease-activated receptor, Mast cell, Tendinopathy

## Abstract

**Background/Aim:**

Tendinopathies are pathological conditions of tissue remodelling occurring in the major tendons of the body, accompanied by excessive nociceptive signalling. Tendinopathies have been shown to exhibit an increase in the number of mast cells, which are capable of releasing histamine, tryptase and other substances upon activation, which may play a role in the development of tendinopathies. This study set out to describe the distribution patterns of a family of receptors called protease-activated receptors (PARs) within the Achilles tendon. These four receptors (PAR1, PAR2, PAR3, PAR4) are activated by proteases, including tryptase released from mast cells, and are involved in fibrosis, hyperalgesia and neovascularisation, which are changes seen in tendinopathies.

**Method:**

In order to study which structures involved in tendinopathy that these proteases can affect, biopsies from patients suffering of mid-portion Achilles tendinosis and healthy controls were collected and examined using immunohistochemistry. Tendon cells were cultured to study in vitro expression patterns.

**Results:**

The findings showed a distribution of PARs inside the tendon tissue proper, and in the paratendinous tissue, with all four being expressed on nerves and vascular structures. Double staining showed co-localisation of PARs with nociceptive fibres expressing substance P. Concerning tenocytes, PAR2, PAR3, and PAR4, were found in both biopsies of tendon tissue and cultured tendon cells.

**Conclusions:**

This study describes the expression patterns of PARs in the mid-portion of the Achilles tendon, which can help explain the tissue changes and increased pain signalling seen in tendinopathies. These findings also show that in-vitro studies of the effects of these receptors are plausible and that PARs are a possible therapeutic target in the future treatment strategies of tendinopathy.

## Introduction

**Figure 1:**
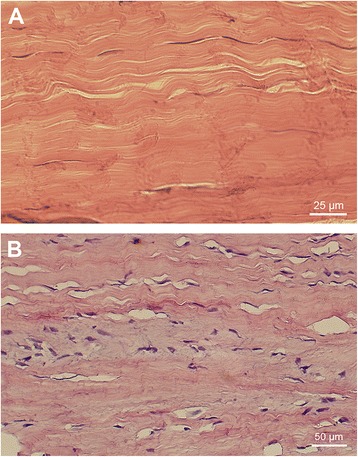
**Histological differences between normal and tendinosis tendon.** The normal tendon shows organised collagen fibres and a sparse amount of tendon cells, tightly packed between the collagen bundles **(A)**. In tendinosis **(B)**, the tendon structure gets disorganised, the tenocytes change morphology and proliferate.

Tendinopathy is a pathological condition of pain and tissue remodelling occurring in the major tendons of the body, such as the Achilles tendon. The condition is commonly thought to originate from overuse of the tendon, as it often afflicts people with a high level of physical activity, although additional aetiological factors have been suggested, such as anatomical predisposition as well as obesity and a lack of physical activity [1-3]. Certain histological traits within the tendon tissue characterize the condition, including hypercellularity, angiogenesis, and increased collagen production [4] (see Figure 1). A histological verified diagnosis with these changes is sometimes called tendinosis [4,5]. This is accompanied by an excessive nociceptive signalling from the tendon, causing pain and restricted mobility [6]. The mechanisms behind these structural and neurological changes are not fully understood. Earlier theories were based primarily on classical inflammation (i.e. “tendinitis”) and collagen damage as the main aetiological factors; however, in later years new hypothesis have arisen, suggesting that other pathways are involved [4,7]. A more recent theory ascribes part of the tendinosis changes to an increased production of biochemical agents, such as substance P (SP), within tendinosis tissue [8].

**Figure 2:**
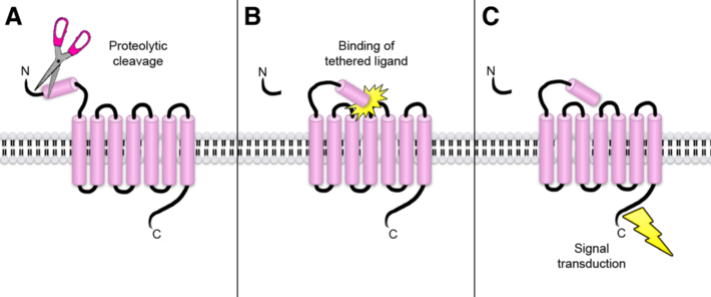
**Activation of protease-activated receptors.** The protease-activated receptors are a family of G-coupled receptors which are activated through proteolytic cleaving **(A)** which unmasks a tethered ligand. This ligand then activates the receptor **(B)** which causes an intracellular signal to be transduced **(C)**. Original art by Gustav Andersson.

Protease-activated receptors (PARs) are a relatively newly discovered group of receptors believed to be involved in the regulation of numerous processes within the human body [9,10]. This family of G protein-coupled receptors consists of four known members (PAR 1–4) that are enzymatically activated by proteolysis of a specific target site on the receptor (see Figure 2). This proteolysis unmasks a tethered ligand attached to the receptor, which in turn activates the receptor [11]. Numerous types of cells have been shown to express PARs such as fibroblasts, neurons, endothelial cells, and mast cells, among others [11]. The involvement of this receptor family has been implicated in the pathophysiology of conditions such as pancreatitis, arthritis, cancer, and lung fibrosis [10,12-15]. Common activators of PARs *in vivo* are trypsin, tryptase, and thrombin [11]. Mast cells contain high amounts of tryptase and, therefore, act as one of the main activators of several PARs *in vivo* [10].

Interestingly, activation of PARs in tissue types other than tendon has been shown to generate several of the characteristic traits that are typical for tendinosis. These changes include fibroblast proliferation [10,14,16-18], angiogenesis [10,19], changes in collagen expression [14,16], and an increased local release of SP [20,21]. Furthermore, a pro-nociceptive effect has been described in which activation of PARs may lead to a sensitization of afferent nerve fibres [13,21-23]. A link between the reported effects of these receptors and the pathology of tendinosis is that recent studies have shown an increase in the number of mast cells in tendinotic tissue and suggest a local effect involved in tendinopathies [24-26]. As tryptase is a known potent activator of PARs [10], degranulating mast cells are possible activators of PARs also concerning tendons.

The possible occurrence and disposition of PARs in relation to tendon tissue is yet to be examined/described. In this study, we therefore aimed to define the expression patterns of PARs on different structures in relation to the Achilles tendon, including the tenocytes themselves–both *in vivo* and in vitro.

## Materials and method

### Biopsies

Tissue biopsies were collected from the Achilles tendon from a total of 26 individuals. Of these, 22 individuals had a documented history of chronic Achilles tendon pain with subsequent impairment of movement. Doppler ultrasound examination showed increased intratendinous blood flow as well as a disorganized collagen structure within the Achilles tendon of these 22 patients, confirming the diagnosis of tendinosis according to established diagnostic criteria [27]. The diagnosis was further established by histological examination following surgery showing hypercellularity, changed tenocyte morphology, and loss of collagen structure within the tendon, indicative of tendinosis [4]. Harvest of the tendinotic biopsies was performed in concert with patients undergoing surgical treatment for their Achilles tendinosis. 3 of the tendinosis patients donating tendon tissue had undergone prior treatments consisting of injections of a sclerosing substance (polidocanol) in the paratendinous tissue surrounding the Achilles tendon. An additional 4 tissue biopsies were collected from healthy individuals volunteering to donate tissue samples. Healthy controls were defined as individuals without history of Achilles tendon pain and/or signs of Achilles tendinosis during Doppler ultrasound examination. All donors were otherwise healthy, non-smokers, and on no medication at the time of surgery.

All tissue samples were collected in strict sterile conditions from the ventral side of the mid-portion of the Achilles tendon (the most affected site in tendinosis patients) using a lateral incision during local anaesthesia. Biopsies were directly placed in sterile saline solution and immediately taken to the laboratory to be prepared for either cell culture or histological staining. Of the 26 biopsies, 21 were chosen for histological sectioning and staining (Table 1) while 5 biopsies were used for cell culture experiments (Table 2). The surgeries, as well as the ultrasound examinations, were performed by Prof H. Alfredson.

**Table 1:** **Patient information of tissue samples used for histological staining**

**Patients n=21**	**Age (years)**	**Sex**	**Diagnosis**	**Prior tendinosis treatment**	**Fixation**
A1	45	Male	Tendinosis	None	No
A2	41	Male	Tendinosis	None	No
A3	59	Female	Tendinosis	None	No
A4	49	Male	Tendinosis	None	No
A5	37	Female	Tendinosis	None	No
A6	68	Female	Tendinosis	None	Yes
A7	44	Female	Tendinosis	None	No
A8	45	Female	Tendinosis	None	No
A9	27	Male	Tendinosis	None	No
A10	34	Male	Tendinosis	None	No
A11	51	Female	Tendinosis	None	No
A12	56	Female	Tendinosis	Polidocanol treatment	No
A13	57	Female	Tendinosis	Polidocanol treatment	No
A14	67	Male	Tendinosis	None	No
A15	45	Female	Tendinosis	None	No
A16	47	Female	Tendinosis	None	No
A17	58	Female	Tendinosis	Polidocanol treatment	No
A18	48	Male	Healthy	None	No
A19	28	Male	Healthy	None	Yes
A20	23	Male	Healthy	None	Yes
A21	21	Male	Healthy	None	Yes

n=21. 10 males, 11 females. Mean age 42.2 years (SD 13.3).

Fixation: treated with formaldehyde (see ‘Histological preparations’).

**Table 2:** **Patient information of tissue samples used for cell culture**

**Patients n=5**	**Age (years)**	**Sex**	**Diagnosis**	**Prior tendinosis treatment**
B1	33	Male	Tendinosis	None
B2	40	Male	Tendinosis	None
B3	55	Male	Tendinosis	None
B4	53	Male	Tendinosis	None
B5	45	Male	Tendinosis	None

n=5. 5 males, 0 females. Mean age 45.2 years (SD 9.1).

The study was approved by the Regional Ethical Review Board in Umeå and was performed according to the principles of the declaration of Helsinki (http://www.epn.se/en/). All donors had given informed consent prior to donation.

### Histological preparations

Biopsies were either fixed by immersion overnight at 4°C, in 4% (w/v) formaldehyde 0.1 M phosphate buffer, pH 7.0, and then washed in the isotonic salt solution Tyrode’s solution (containing 10% (w/v) sucrose) before being mounted on thin cardboard using OCT embedding medium (Miles Laboratories, Naperville, IL, USA) and snap-frozen in liquid nitrogen chilled propane, or immediately frozen without fixation. Samples were then stored at–80°C prior to sectioning and staining. Sectioning was done using a cryostat producing sections of 7 μm thickness, which were mounted on chrome-alum-gelatine pre-treated slides. Notably, a few biopsies consisted of insufficient amount of tissue to provide sections to stain all four PAR-receptors (PAR-1 n=20, PAR-2 n=20, PAR-3 n=19, PAR-4 n=19).

Sections were stained according to an established protocol [28]. Four polyclonal rabbit antibodies specific for each respective PAR receptor were used, and a monoclonal mouse antibody for SP, all at concentrations of 1:100 (see Table 3). The frozen sections were initially allowed to thaw for 10 minutes at room temperature. After this the samples were submerged in 1% (v/v) triton X-100 solution for 20 minutes for permeabilisation, followed by 3x5min washing in phosphate buffered saline (PBS). Incubation with swine normal serum was performed at a concentration of 1:20 for 15 minutes prior to an overnight incubation with the primary antibody at 4°C. The sections were then washed for an additional 3x5min in PBS followed by a second incubation with swine normal serum 1:20 for 15 minutes. This was followed by an incubation with a secondary swine anti-rabbit antibody conjugated with tetramethylrhodamine-5-(and 6)-isothiocyanate (TRITC) (Dako, Copenhagen DN; R0156), for fluorescent detection, at a concentration of 1:40 for 30 minutes at 37°C. Finally, a series of washes with PBS as mentioned in previous text was performed followed by mounting of the sections using Vectashield (Vector Labs, Burlingame, CA, USA; H-1200) containing 4’,6-diamidino-2-phenylindole (DAPI) for nuclear counter staining. Control stainings were performed by replacing the primary antibody with PBS to control for unspecific antigen binding of the secondary antibody.

**Table 3:** **Antibodies used for detection of PAR 1–4 and Substance P**

**Target**	**Code**	**Source**	**Raised in**	**Concentrations used**
PAR-1	APR-031	Alomone Labs, Jerusalem, Israel	Rabbit	1:100
PAR-2	APR-032	Alomone Labs, Jerusalem, Israel	Rabbit	1:100
PAR-3	ab66068	Abcam, Cambridge UK	Rabbit	1:100
PAR-4	ab66103	Abcam, Cambridge UK	Rabbit	1:100
Substance P	8450-0505	AbD Serotec, Oxford, UK	Mouse	1:100

Double staining with SP involved additional steps of washing in PBS following the incubation of the secondary antibody towards the PAR-antibody, as described above, and incubation in donkey normal serum 1:20 for 15 minutes, and then incubation with the primary SP antibody overnight at 4°C. The subsequent steps where then repeated as for the solitary PAR-staining described above, with the supplementation of swine normal serum with donkey normal serum and the secondary antibody with a donkey anti-mouse antibody conjugated with 1:500 Alexa Fluor® 488 dye (Invitrogen, CA, USA: A-21202).

### Histological evaluation

All sections were inspected using a Zeiss Axioscop 2 Plus microscope equipped with epifluorescence and an Olympus DP70 digital camera. Expression of PAR-1,-2,-3 and–4 was subjectively evaluated by two observers (JC, GA) independently of each other. The examiners were blinded as to whether the biopsies came from healthy or tendinotic tendons, however, the receptor evaluated was not blinded for. Three specific structures of interest within the biopsies–tenocytes, vessels, and nerves–were selected due to their involvement in tendinosis pathology [4,27], and given a semi-quantitative score of 0–3 based on the intensity of fluorescence and amount of reactive structure. Not all biopsies displayed the specific structures previously mentioned and in these cases they were not given a score for that structure. All samples were photographed for documentation. The results from each observer were pooled together yielding a mean score for each PAR-receptor in the respective tissue structure of interest. Evaluation of co-localisation of PARs and SP was performed to see if PARs are expressed on nociceptive fibres in the tendon related tissues. No immunohistochemical staining was performed to differentiate tenocytes or vessels in this study. Parallel sections stained with H&E were used to identify structures in cases of uncertainty.

### Human tendon cell culture

Human Achilles tendon biopsies were initially washed in Hanks balanced salt solution (HBSS) and, by means of a scalpel, cleared of any visible tissue that was not of the tendon tissue proper. The biopsies were then manually divided into smaller pieces and enzymatically digested using a collagenase solution (Clostridopeptidase A, C-0130 Sigma) diluted in D-MEM (Invitrogen; 11960) at a concentration of 2 mg/ml. The product was centrifuged to obtain a cell pellet and supernatant. The supernatant was discarded and the cell pellet was washed in HBSS and later dissolved in media consisting of Dulbecco’s Modified Eagle Medium (D-MEM) supplemented with 10% (v/v) foetal bovine serum (FBS), 1% (v/v) pen-strep antibiotics and 0.2% (v/v) L-Glutamine. This solution was portioned out in cell culture flasks and stored in a humidified environment at 37°C/5% (v/v) CO_2_. Media was replaced every 72 h. As soon as cells reached confluence in the culture flasks they were passaged and split into new flasks in a 1:3 ratio. Cells were used for immunohistochemical stainings at passage 3–5 by seeding them on glass slides over night. The immunohistochemical staining was carried out in the same manner as described under ‘Histological preparations’, following a 10 min fixation in 3% (v/v) paraformaldehyde. This protocol has been shown to produce a tendon cell phenotype, as verified by earlier studies [29].

## Results

### Histological appearance

The biopsies taken from tendinosis patients displayed classical signs of tendinosis: hypercellularity, changed cell morphology, and loss of tissue structure, as well as signs of newly formed vessels in the tendon tissue itself. Not all structures of interest were featured in every biopsy. This can be derived from the fact that therapeutic surgery on Achilles tendinosis consists of scraping of the ventral border of the tendon, sometimes resulting in biopsies with a scarce amount of tendon tissue proper and varying amounts of paratenon and other paratendinous structures.

Biopsies from healthy donors displayed morphology of normal tendon tissue proper and paratenon, i.e. no hypercellularity, normal tenocyte morphology, and normal tendon tissue structure. No evident vascular changes were seen in these biopsies.

### Incidence and disposition of PAR 1–4

No statistical significant differences were seen between the healthy controls and the tendinosis tendons concerning the degree of PAR-expression in the different structures of interest when subjecting the data to non-parametric statistical tests. The results of the immunohistochemical evaluations are presented in Table 4.

**Figure 3:**
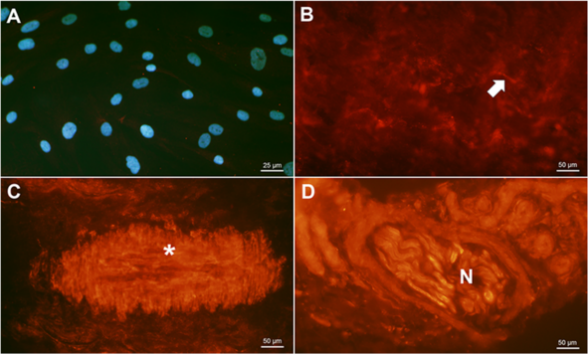
**PAR-1 expression in tendon tissue and cultured cells.** PAR-1 immunostaining (red) was not found in tenocytes (arrow) in either tissue sections from tendon biopsies **(B)** or in cultured cells **(A)**. DAPI-combined-staining (blue) is shown in **(A)** to visualize cells without reactive staining for PAR-1. Any red staining in **(A)** and **(B)** is considered background staining and not specific reactions. Vessels (asterisk in **C**) and nerve fibres (N) in **(D)** were clearly reactive. **B** is a healthy tendon section, **C** & **D** are from tendinosis.

PAR-1 was not expressed on tenocytes, in tissue sections or in cultured tenocytes (Figure 3A and B), on any of the slides investigated. The receptor was detected at relatively low level on vessels (Figure 3C, mean score of normal tendons (MSn): 1.6, mean score of tendinosis tendons (MSt): 1.0), but was frequently expressed in nerve fibres (Figure 3D, MSn: 3.0, MSt: 2.2).

**Figure 4:**
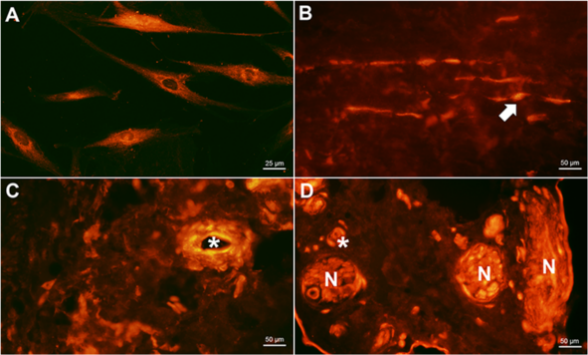
**PAR-2 expression in tendon tissue and cultured cells.** Positive immunostaining for PAR-2 (red) in cultured tenocytes **(A)** vessels (*asterisk* in **C** & **D**), and nerve fibres (N in **D**), as well as in tendon biopsies (*arrow* in **B**). All sections **(B-D)** are from healthy tendons.

PAR-2 was detected on tenocytes both in vitro (Figure 4A) and in biopsies (Figure 4B, MSn: 2.7, MSt: 2.8). It was also expressed in some nerves (Figure 4D, MSn: 1.8, MSt: 1.5) and vessels (Figure 4C, MSn: 2.3, MSt: 1.8). This suggests a presence of PAR-2 in all three structures of interest, but the strongest reactions were seen in the tenocytes themselves. Concerning nerves, they were quite scarcely expressed, and only a fraction of the samples showed clear immunofluorescence when the nerves were stained for PAR-2 (cf. Table 4).

**Table 4:** **Scores from evaluation of tendon biopsies**

	**NORMAL TENDONS (n=4)**				**TENDINOSIS TENDONS (n=17)**			
	**PAR-1**	**PAR-2**	**PAR-3**	**PAR-4**	**PAR-1**	**PAR-2**	**PAR-3**	**PAR-4**
	Tenocytes				Tenocytes			
n	3	3	3	3	12	12	11	11
Mean	0.0	**2.7**	2.5	1.3	0.0	**2.8**	1.4	1.5
SD	0.0	0.3	0	0.3	0.0	0.45	0.6	0.7
Median	0.0	2.5	2.5	1.5	0.0	3.0	1.5	1.5
IQR	0.0	2.5-2.8	2.5-2.5	1.3-1.5	0.0	2.9-3.0	1.0-1.8	1.0-1.75
	Vessels				Vessels			
n	4	4	4	4	14	12	15	15
Mean	1.6	2.3	**2.3**	**2.8**	1.0	1.8	**2.2**	**2.4**
SD	0.3	0.3	0.3	0.5	0.4	0.6	0.3	0.4
Median	1.5	2.3	2.3	3.0	1.5	2.0	2.0	2.5
IQR	1.5-1.6	2-2.5	2.0-2.5	2.8-3.0	1.5-1.6	1.5-2.0	2.0-2.5	2.0-2.5
	Nerves				Nerves			
n	3	2	4	4	9	4	2	10
Mean	**3.0**	1.8	1.5	2.4	**2.2**	1.5	1.0	**2.4**
SD	0.0	0.4	0.6	0.5	0.8	0.6	0.0	0.4
Median	3.0	1.8	1.5	2.3	2.0	1.5	1.0	2.3
IQR	2.0-3.0	1.6-1.9	1.0-2.0	2.0-2.6	2.0-3.0	1.0-2.0	1.0-1.0	2.0-2.5

n: number of samples containing structure of interest, SD: Standard deviation, IQR: interquartile range. Bold number represent most prominent reactive structure for each receptor.

**Figure 5:**
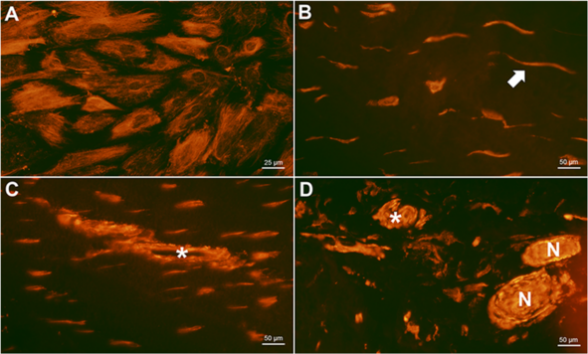
**PAR-3 expression in tendon tissue and cultured cells.** Positive reactions for PAR-3 (red) shown in cultured tenocytes **(A)**, tenocytes in biopsies (*arrow* in **B**), vessels (*asterisk* in **C** & **D**), as well as in nerve fibres (N in **D**). **B** & **C** are sections showing tendinosis, **D** is from a healthy tendon.

PAR-3 also stained positive on tenocytes in vitro (Figure 5A) and in biopsies (Figure 5B, MSn: 2.5, MSt: 1.4), but similar to PAR-2, only some nerves (Figure 5D, MSn: 1.5, MSt: 1.0) were reactive. The receptor was most evident in vessels (Figure 5C, MSn: 2.3, MSt: 2.2).

**Figure 6:**
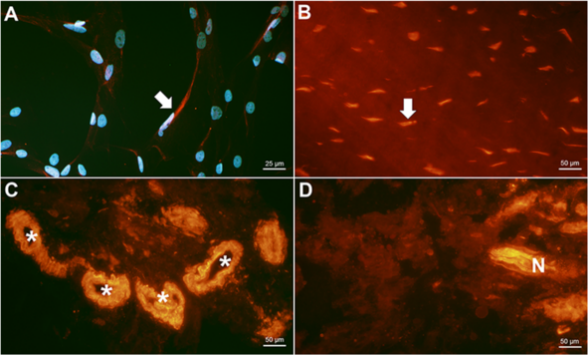
**PAR-4 expression in tendon tissue and cultured cells.** PAR-4 stainings showing positive results (red) on tenocytes in culture (*arrow* in **A**), tenocytes *in vivo* (*arrow* in **B**), vessels (*asterisk* in **C**), and nerve fibres (N in **D**). DAPI-combined-staining is shown in **A** to visualize cells with weak staining for PAR-4 in close relation to nuclei. All sections **(B-D)** are from tendinosis tendons.

Lastly, PAR-4 displayed a staining pattern similar to that of PAR-2 and–3, staining positively on tenocytes in vitro (Figure 6A) and in biopsies (Figure 6B, MSn: 1.3, MSt: 1.5). The staining of nerves (Figure 6D, MSn: 2.4, MSt: 2.4) and vessels (Figure 6C, MSn: 2.8, MSt: 2.4) was stronger than seen for the tenocytes. The staining pattern found on tenocytes for PAR-4 appeared to be not only weaker but also in closer relation to the nucleus as compared to PAR-2 and–3, which showed a homogeneous staining of the whole tenocyte.

**Figure 7:**
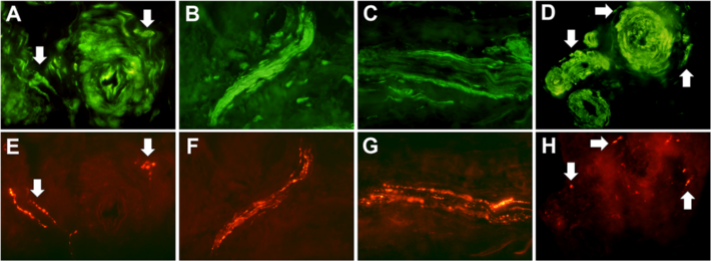
**Double stainings for PARs and substance P. A**-**D** shows PARs [1-4] and **E**-**H** shows corresponding SP-staining; PAR-1 **(A)** is co-localised with SP-positive nerve fibres **(E)** (*arrows*). The same was seen for PAR-2 (nerve fascicle in **B**&**F**), PAR-3 (nerve fascicle in **C**&**G**) and PAR-4 (**D**&**H**, arrows show perivascular nerve fibres).

Double stainings of the PAR-receptors and SP showed that nerve fibres and fascicles expressing the PAR-receptors often co-localised with SP (Figure 7A-H). Not all the nerve fibres expressing PARs were positive for SP.

## Discussion

Our study shows that all the four protease-activated receptors are expressed in human tendon tissue to varying levels and distributions of expression. Nerve fibres expressing PARs were co-localised with SP–a marker for nociceptive fibres–but were also expressed on nerve fibres not showing SP-positive reactions. This shows that other nerves than nociceptive ones may be regulated by PAR-activation. The expression pattern and intensity seems to be similar in tendinosis tendon tissue and in healthy tendons, although this study lacked sufficient biopsies from healthy donors to make an adequate comparison between the groups. It is important to note that the technique of immunofluorescence employed in this study cannot be used to compare the amount of the individual receptors against each other, but only when comparing the localisation of the same receptor in the same sample. However, the extent to which the receptors showed immunofluorescence in the different tissue types studied (tenocytes, nerves and vessels) can hint at where they have the most effect. Of note is also that only 6 out of the 21 biopsies studied showed evident nerve fibre reactions for PAR2 and PAR3, which could be explained by the specific sections actually not containing any nerves. Perhaps these receptors are not expressed on all kinds of nerves, but this study shows a co-localisation on SP-positive fibres, which are considered nociceptive.

## Conclusions

PAR-1 was mainly found on nerves and to a lesser extent on vessels within tendon tissue. The receptor was not seen on tenocytes, indicating that this receptor does not have a direct effect on these cells. In a study on breast cancer, PAR-1 was differentially expressed on stromal fibroblasts and in the healthy fibroblasts the receptor was not expressed whereas activated fibroblast started to express the receptor [30]. PAR-1 agonists can cause arterial constriction [31] and thus control blood flow, but has also been connected to angiogenesis [32]. Other studies have shown that PAR-1 is involved in sensitising nociceptive neurons [33], pain signalling and neurogenic inflammation [11,34,35] by direct effect on nerve fibres, functions that could be of importance in tendinosis pathology as neurogenic inflammation has been implied as one of the contributors to tendinopathies [36]. On the other hand, a study be Martin et al. [37] found that the effects of PAR-1 stimulation caused an inhibition of inflammatory pain through activation of endogenous opioid pathways in the peripheral tissue itself. If the same can be seen in tendon tissues, this could potentially give rise to localised treatments with PAR-1 agonists as pain relief in tendinosis patients.

PAR-2 is known to be involved in many processes similar to those that occur in tendinosis, in other tissues. These processes include fibroblast proliferation [10,14,16], angiogenesis [10,19], hyperalgesia [23], changes in collagen expression [14,16], and an increased, local release of SP [20,21]. In our study, PAR-2 was identified within all three cell-types/structures of interest. Since this receptor is activated by tryptase, a protease abundant in mast cells, and there is a known increase in mast cell numbers in tendinosis tendon tissue [24], the discovery of PAR-2 expression within tendon tissue makes it logical to presume that this receptor can play a role in tendon pathology. The PAR-2 expression on vascular structures could be linked to a possible role in the angiogenesis described for tendinopathy, as other studies have shown that PAR-2 agonists can drive angiogenesis in an in-vivo ischemia model [38]. Studies have shown PAR2 to be co-localised with TRPV1 receptors and cause hyperalgesia [39].

PAR-3 was found on all three cell-types/structures of interest. Unlike PAR-1,–2 and–4, there are few known effects of PAR-3 stimulation other than that it acts as a co-receptor for PAR-4 [11,40].

PAR-4 was also found on nerves, vessels and tenocytes. This receptor has mainly been implicated in pain modulation by direct effect on nerve fibres where it seems to be able to sensitize as well as desensitize nerve fibres depending on the environment in which the receptor is activated [22,33,41]. This pain modulating effect could be of importance in tendinosis development, considering that increased tendon pain is one of the cardinal symptoms of the condition.

It should be clarified that the reactions for nerves and vessels were seen predominantly in the tissues outside of the tendon tissue proper. These structures have been described earlier [42] and are targeted in novel treatment strategies directed at the ventral side of the Achilles tendon, such as ultrasound-guided scraping [27,43,44]. This fact may have caused an undersampling of the neuronal PAR expression in this study, as most biopsies contained tendon tissue proper and only smaller parts of the paratendinous tissues, such as; epitenon, paratenon and the loose connective tissue ventral to the tendon. Future studies on neuronal PAR expression may want to focus on these tissues as the tendon tissue proper is mostly devoid of nerve fibres [45].

**Figure 8:**
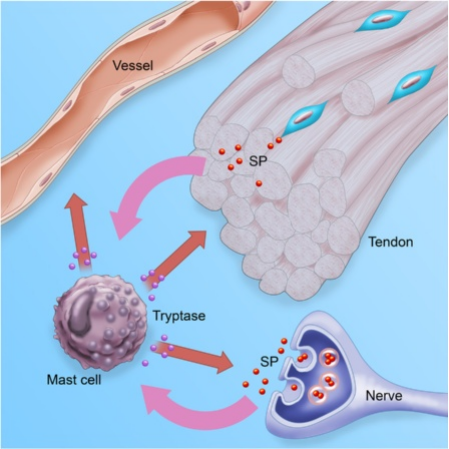
**Interaction of mast cell with tissues involved in tendinosis.** Mast cells are recruited to the tendon tissue in cases of tendinosis, the mechanism behind which is unclear. A possible attractant is neuropeptides, such as SP (*red dots*), which can be produced by the tenocytes or released from peripheral nerve endings. These neuropeptides are also capable of causing the mast cells to degranulate, where upon tryptase and other proteases (*blue dots*) can affect the tendon, vessels and nerves via the protease-activated receptors. Original art by Gustav Andersson.

As was stated in the introduction, tendon cells have been shown to produce and release SP [8,28], a neuropeptide involved in pain-transmission and neurogenic inflammation [46]. It is also known that SP can attract mast cells and cause them to degranulate [47,48]. A part in the “biochemical” hypothesis for tendinopathy could therefore include the interplay between locally produced neuropeptides, such as SP, and the attraction and degranulation of mast cells (see Figure 8).

As this study has shown that tenocytes in culture have similar expression patterns of PARs as they do *in vivo*, it is possible to study the effects of PAR-activation on tenocytes in an in vitro setting. By better understanding the mast cell and PAR interaction in tendinopathies, treatment of these conditions could come to include mast cell stabilizing agents such as over-the-counter allergy medicines, or directed PAR agonists and antagonists which are under development [49,50]; both of which could become important therapeutic tools.

In summary, protease-activated receptors are expressed in the Achilles tendon and surrounding tissues. PAR-1 and–4 was found most frequently in nerves, whereas PAR-2 was expressed primarily by tenocytes. All four PARs where expressed on vessels in and around the tendon. All four PARs co-localised with SP-positive nerve fibres. More control samples are needed to study whether healthy and tendinosis tendons display varying degrees of PAR expression, as no significant difference was seen in this study. Further studies are needed to decide what effects the activation of the individual receptors may have in the development and possible treatment of tendinosis.
